# Calcium Phosphate‐Coated and Strontium‐Incorporated Mesoporous Silica Nanoparticles Can Effectively Induce Osteogenic Stem Cell Differentiation

**DOI:** 10.1002/adhm.202101588

**Published:** 2021-11-25

**Authors:** Pichaporn Sutthavas, Zeinab Tahmasebi Birgani, Pamela Habibovic, Sabine van Rijt

**Affiliations:** ^1^ Department of Instructive Biomaterials Engineering MERLN Institute for Technology‐Inspired Regenerative Medicine Maastricht University P.O. Box 616 Maastricht 6200 MD the Netherlands

**Keywords:** bioinorganics, bone regeneration, calcium phosphate, mesoporous silica nanoparticles, osteogenesis

## Abstract

Ceramic (nano)materials are promising materials for bone regeneration applications. The addition of bioinorganics such as strontium (Sr) and zinc (Zn) is a popular approach to further improve their biological performance. However, control over ion delivery is important to prevent off‐target effects. Mesoporous silica nanoparticles (MSNs) are popular nanomaterials that can be designed to incorporate and controllably deliver multiple ions to steer specific regenerative processes. In this work, MSNs loaded with Sr (MSN_Sr_) and surface coated with a pH‐sensitive calcium phosphate (MSN_Sr_‐CaP) or calcium phosphate zinc layer (MSN_Sr_‐CaZnP) are developed. The ability of the MSNs to promote osteogenesis in human mesenchymal stromal cells (hMSCs) under basic cell culture conditions is explored and compared to ion administration directly to the cell culture media. Here, it is shown that MSN‐CaPs can effectively induce alkaline phosphatase (ALP) levels and osteogenic gene expression in the absence of other osteogenic stimulants, where an improved effect is observed for MSNs surface coated with multiple ions. Moreover, comparatively lower ion doses are needed when using MSNs as delivery vehicles compared to direct ion administration in the medium. In summary, the MSNs developed here represent promising vehicles to deliver (multiple) bioinorganics and promote hMSC osteogenesis in basic conditions.

## Graphical abstract

Mesoporous silica nanoparticles (MSNs) loaded with strontium ions (MSN_Sr_) and surface coated with a pH‐sensitive calcium phosphate (MSN_Sr_‐CaP) or calcium phosphate zinc layer (MSN_Sr_‐CaZnP) are reported. It is demonstrated that the developed MSNs are taken up by human mesenchymal stromal cells and can efficiently promote osteogenesis under basic conditions, especially when multiple ions are introduced.

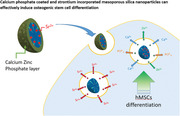

## 1 Introduction

There is a high demand for bone substitutes due to traumatic injury, bone disease, and an increasingly ageing population. The gold standard treatment is the use of patients own bone (i.e., autografts). However, drawbacks associated with autografts including limited availability have led to the development of synthetic bone substitutes. Inorganic materials prepared from calcium (Ca), phosphate (P), and silica (Si), such as ceramics and bioactive glasses, are considered promising candidates for bone regeneration applications due to their inherent bioactivity and chemical similarity to natural bone.^[^ ^1^ ^]^ The biological performance of ceramic materials can be further improved by using additives such as growth factors, small molecules, and bioinorganic ions.^[^ ^2^ ^]^ Especially, bioinorganic ions are considered interesting candidates as they are known to play important roles in bone formation processes.^[^ ^3^ ^]^ For example, strontium (Sr), chemically and physically similar to Ca, is known to promote the activity of osteoblastic cells, while inhibiting osteoclast activity.^[^ ^4^ ^]^ Another example of a popular additive is zinc (Zn), which is an essential trace element important in many cellular processes, including bone remodeling by supporting both pre‐osteoblast differentiation and suppressing osteoclast differentiation.^[^ ^5^ ^]^ The therapeutic use of bioinorganics such as Sr and Zn is promising as they are biocompatible, nonimmunogenic, and are easily taken up by cells via ion channels and membrane diffusion. However, this capability to be rapidly processed by cells can also lead to nonspecific adverse effects if distributed systemically without dosage control. For example, in a study by Yu et al. it was demonstrated that Zn can have a double‐edged effect; Zn doses between 2 and 5 µg mL^−1^ stimulated mesenchymal stromal cell (MSC) adhesion and proliferation, however, higher Zn doses of 15 µg mL^−1^ led to inhibition of osteogenic differentiation and induced MSCs apoptosis.^[^ ^6^ ^]^ As such, the route of administration and dosage scheme are important parameters to be taken into account when considering the therapeutic use of bioinorganics. Ion substitution within the main network of ceramics is a popular method to incorporate and release bioinorganic ions and resulted in scaffolds with improved bone regenerative performance.^[^ ^7^ ^]^ Although the effect of combining ions in a single construct has only limitedly been explored, several studies have shown that incorporating multiple ions into calcium phosphate scaffolds improves their bone formation capabilities compared to scaffolds without additional ions.^[^ ^8^ ^]^ However, ion substitution within ceramics can alter the mechanical properties and degradation rates, which compromises their structure and in addition makes it difficult to precisely control ion release.

Mesoporous silica nanoparticles (MSNs) are popular carriers for biomolecule delivery, and have emerged as promising materials for use in bone regeneration applications.^[^ ^9^ ^]^ MSNs unique characteristics such as high drug‐loading capacity, large surface area, and high pore volume as well as tunable pore and particle size make them flexible tools to optimize cargo delivery. Many approaches have been reported where MSNs have been used for the effective delivery of growth factors, ions, genetic material, and antibiotics.^[^ ^10^ ^]^ MSNs surface chemistry can, in addition, be easily modified with capping agents to allow controlled cargo delivery, e.g., in response to pH, temperature, or light.^[^ ^11^ ^]^ MSNs represent a particularly interesting alternative strategy for bioinorganic ion delivery because multiple therapeutic ions can be integrated in one nanoparticle construct, e.g., within the silica matrix, mesopores, or grafted onto the surface, resulting in unique ion release profiles. Moreover, MSNs can easily be incorporated in other biomaterials and scaffolds,^[^ ^12^ ^]^ and as such are a promising strategy to improve ion delivery without impairing material characteristics. Finally, MSNs are processed differently by cells compared to inorganic ions; where ions are taken up via ion channels present on the cell membrane, MSNs are internalized via endocytotic pathways.^[^ ^13^ ^]^ We hypothesize that ion delivery using MSNs is more efficient compared to direct bioinorganic ion administration in cell culture media.

In this work, we developed MSNs loaded with Sr and coated with CaP or CaZnP layer as efficient and versatile carrier systems for multiple bioinorganic ion delivery to human mesenchymal stromal cells (hMSCs). Here, Zn and Sr were chosen due to their known positive effects on the osteogenic differentiation of hMSCs, however the design allows facile encapsulation of other ions. CaP surface functionalization was chosen as both a pH‐sensitive gating system and to simultaneously deliver Ca and P to hMSCs, which play important roles in stimulating bone formation.^[^ ^1^ ^]^ Cell internalization and the ability of the synthesized MSNs to promote osteogenesis in hMSCs under basic cell culture conditions was explored and compared to ion administration directly to the cell culture media.

Here, we show that MSNs incorporating Sr, and coated with Ca, P, and Zn can effectively induce osteogenic marker expression in the absence of other osteogenic stimulants, where an improved effect was observed for MSNs functionalized with multiple ions. Moreover, comparatively lower ion doses were needed when using MSNs as delivery vehicles compared to direct ion administration in the cell culture media. In summary, the MSNs developed here represent promising vehicles to efficiently and locally deliver bioinorganics and promote hMSC osteogenesis in basic conditions.

## 2 Results

### 2.1 Synthesis and Characterization of Sr‐Loaded and Ca, P, Zn‐Surface Coated MSNs

**Figure 1:**
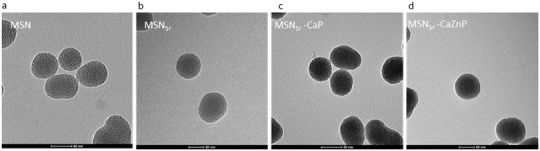
Characterization of a) MSN, b) MSN_Sr_, c) MSN_Sr_‐CaP, and d) MSN_Sr_‐CaZnP by TEM. Scale bar is 50 nm.

To allow ion incorporation, MSNs with multiple core‐shell modifications were prepared. In the first step, MSNs functionalized with thiols in the core structure were synthesized via hydrolysis and condensation of silica precursors in the presence of a micelle template, as we have reported previously.^[^ ^14^ ^]^ The presence of the thiol groups was confirmed by labeling with a thiol reactive fluorescent dye (ATTO‐647‐maleimide; Figure S1, Supporting Information). The surface and mesopores of the MSNs were then further modified with amine groups using 3‐aminopropyl triethoxysilane (APTES) post‐grafting to create MSN‐NH_2_. Spherical, evenly shaped MSNs with a mesoporous structure were obtained (Figure S2a, Supporting Information). In addition, the surface zeta potential changed from negative (−20.6 ± 0.1 for MSNs) to a positive zeta potential (19.6 ± 0.3 for MSN‐NH_2_), after amine surface grafting (Table S1, Supporting Information). The surface‐grafted amines could then be further modified by reaction with succinic anhydride to carboxylic acid groups (MSN‐COOH), to allow Ca and P deposition on the surface of MSNs., as we reported previously.^[^ ^15^ ^]^ The surface modification resulted in another surface potential change to −17.7 ± 0.2, indicating that the modification from positively charged amine groups to negatively charged carboxylic acid groups was successful (Table S1, Supporting Information). The carboxylic acid group modification was further confirmed by Fourier transform infrared spectroscopy with an observable peak of around 1627 cm^−1^ (COOH stretch, Figure S2c, Supporting Information). Two types of MSN‐COOH particles were created; one with COOH groups only on the nanoparticle surface and one where COOH groups were incorporated on the surface and in the mesopores. The COOH‐modified MSNs were then soaked in a Sr solution to create MSN_Sr_. The MSNs containing COOH in the mesopores showed increased uptake of Sr ions compared to the MSNs which only had COOH groups on the surface (Figure S1, Supporting Information), and were used for the remainder of the study. Sr incorporation did not alter the morphology of the MSNs (**Figure** **1**b).

To graft the CaP layer on the surface of the MSNs to create MSN_Sr_‐CaP, Ca and P precursors in solution were added alternately on MSN_Sr_ until a thin layer of CaP was obtained (Figure 1b). To additionally add Zn to the CaP layer, Zn precursors were added along with Ca precursors during layer formation at 10% molar equivalent, to create MSNs with a thin layer of Zn‐doped CaP (MSN_Sr_‐CaZnP, Figure 1c). The X‐ray diffraction (XRD) pattern of MSNs grafted with Ca and P in the absence of Sr and Zn ions (MSN‐CaP) showed only broad peaks, suggesting the formation of a rather amorphous CaP layer using this method. Similarly, the XRD analysis indicated that the CaP layer on MSN_Sr_‐CaP was amorphous and that MSN_Sr_‐CaZnP had a low of crystallinity (Figure S3, Supporting Information). Inductively coupled plasma mass spectrometry (ICP‐MS) analysis of the dissolved nanoparticles confirmed the presence of Ca, P, Sr, and Zn in the MSNs (**Table** **1**). Furthermore, MSN_Sr_, MSN_Sr_‐CaP, and MSN_Sr_‐CaZnP were similar in size, with hydrodynamic sizes of 203 ± 5, 218 ± 14, and 223 ± 10 nm, respectively (**Table** **2**). The nanoparticles were monodispersed with polydispersity indexes (Pdi) of 0.1 (MSN_Sr_), 0.33 (MSN_Sr_ ‐CaP), and 0.27 (MSN_Sr_‐CaZnP) and had a negative surface potential (Table 2). In summary, our layer‐by‐layer method enabled the formation of MSNs incorporating Sr and coated with Ca, P, and Zn, which were homogenous in both size and shape.

**Table 1:** Ion composition of MSN_Sr_, MSN_Sr_‐CaP, and MSN_Sr_‐CaZnP (in 100 mg) using ICP‐MS analysis

Sample	Si [mg]	Ca [mg]	P [mg]	Zn [mg]	Sr [mg]
MSN_Sr_	99.97613	–	–	–	0.03856
MSN_Sr_‐CaP	95.57849	2.759965	1.652674	–	0.04526
MSN_Sr_‐CaZnP	95.41935	2.700724	1.626942	0.248765	0.04222

**Table 2:** Hydrodynamic size, Pdi, and surface zeta potential of MSN_Sr_, MSN_Sr_‐CaP, and MSN_Sr_‐CaZnP in ethanol measured by dynamic light scattering

Sample	Hydrodynamic size in ethanol [nm]	Pdi	Zeta potential
MSN_Sr_	203 ± 5	0.1	−5.34
MSN_Sr_‐CaP	218 ± 14	0.33	−20.43
MSN_Sr_‐CaZnP	223 ± 10	0.27	−20.56

### 2.2 Ion Release from MSNs at Neutral and Acidic pH

**Figure 2:**
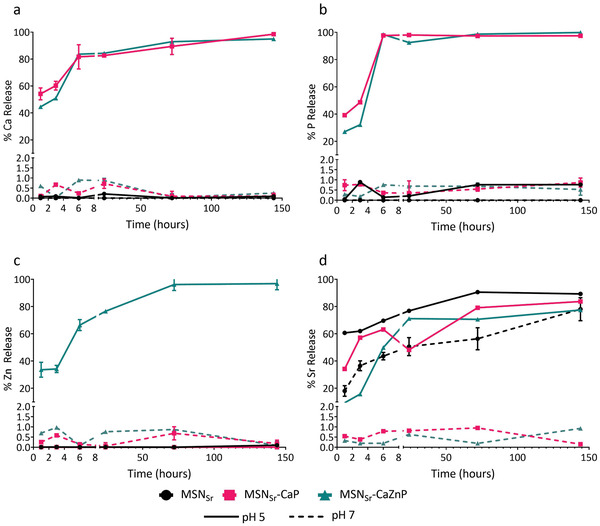
MSN ion release in neutral and acidic pH. Ion release profiles of a) Ca, b) P, c) Zn, and d) Sr from MSN_Sr_ (black), MSN_Sr_‐CaP (red), and MSN_Sr_‐CaZnP (blue) in cacodylate buffer pH 5 (solid lines) and pH 7.4 (dashed lines) analyzed by ICP‐MS. The percentage of each element present in the supernatant compared to total amount is shown.

To assess ion release at different pH found in extracellular (pH 7.4) and intracellular (endosomes; pH 5) environments, MSN_Sr_, MSN_Sr_‐CaP, and MSN_Sr_‐CaZnP were incubated in cacodylate buffer (does not contain Ca, P, Zn, Sr, or Si), at pH 5 and 7.4 for 0, 1, 3, 6, 24, 72, and 144 h, and ion release was quantified using ICP‐MS (**Figure** **2**).

MSN_Sr_‐CaP and MSN_Sr_‐CaZnP were stable at neutral pH as no significant Si, Ca, P, or Zn release could be observed after 6 days of incubation (Figure 2). Also no Sr release was observed from MSN_Sr_‐CaP or MSN_Sr_‐CaZnP at pH 7.4. In contrast, Sr diffused out rapidly from nongated nanoparticles (MSN_Sr_) with 43.6% release after 6 h in neutral pH.

At pH 5, rapid dissolution of the CaP layer was observed. Specifically, the calcium phosphate layer of MSN_Sr_‐CaP and MSN_Sr_‐CaZnP showed total dissolution of P within 6 h (Figure 2a,b). Interestingly, Ca and Zn release was slower than that of P (Figure 2c), which might be due to ion adsorption on to the MSNs. Sr release at pH 5 was similar for all three nanoparticles, and slower compared to Zn and Ca degradation, this may be due to increased affinity of Sr toward carboxylic acid groups present in the MSN mesopores (Figure 2a,c). The MSN matrix remained stable for all nanoparticles at both pH 7.4 and pH 5 (Figure S4, Supporting Information).

In summary, here we showed that the CaP surface coating on MSNs prevented release of encapsulated and surface‐coated ions when in neutral (extracellular) pH. The CaP and CaZnP surface coatings could rapidly degrade at acidic pH values like those found in endosomes/lysosomes, triggering ion release of the surface‐coated and encapsulated ions, where the mesoporous silica matrix remained intact.

### 2.3 Biocompatibility and Cell Internalization

**Figure 3:**
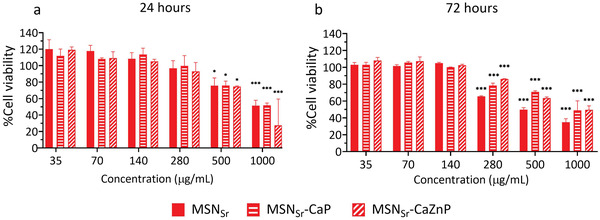
hMSC metabolic activity of MSN_Sr_, MSN_Sr_‐CaP, and MSN_Sr_‐CaZnP at concentrations ranging from 35 to 1000 µg mL^−1^ after a) 24 and b) 72 h of exposure using the MTT assay. Cell viability is shown as % compared to control cells (not incubated with MSNs). Significant differences between sample groups and control groups are shown by * representing *p*‐values as follows; *p < 0.033; **p < 0.02; ***p < 0.001.

Next, the biocompatibility of MSN_Sr_, MSN_Sr_‐CaP, and MSN_Sr_‐CaZnP at concentrations ranging from 35 to 1000 µg mL^−1^ was assessed in hMSCs using the MTT (3‐(4,5‐dimethylthiazol‐2‐yl)‐2,5‐diphenyl tetrazolium bromide) assay. Only relatively high nanoparticle concentrations of 500 and 1000 µg mL^−1^ MSN_Sr_, MSN_Sr_‐CaP, and MSN_Sr_‐CaZnP led to a significant decrease in hMSC cell metabolism after 24 h of exposure compared to control cells cultured in the absence of MSNs (**Figure** **3**a). After 3 days of exposure, also the lower concentration of 280 µg mL^−1^ resulted in a significant decrease in cell metabolism for all three tested MSNs (Figure 3b). There was no statistical difference between the MSN groups after 24 h, however, after 72 h, MSN_Sr_ was significantly more toxic than MSN_Sr_‐CaP and MSN_Sr_‐CaZnP at the higher concentration range (280–1000 µg mL^−1^). In conclusion, MSN concentrations lower or equal to 140 µg mL^−1^ did not affect hMSCs viability for any of the MSNs tested. Based on these results, 70 and 140 µg mL^−1^ were selected for further studies.

**Figure 4:**
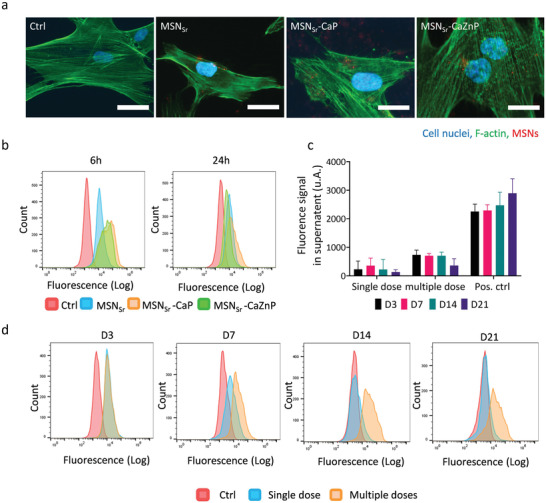
MSN internalization in hMSCs. a) Fluorescence microscopy images of hMSCs exposed to MSN_Sr_, MSN_Sr_‐CaP, and MSN_Sr_‐CaZnP at 140 µg mL^−1^ for 6 h. Cells stained for nuclei (DAPI; blue) and actin (phalloidin 488; green). MSNs are shown in red. Scale bars are 50 µm. b) Flow cytometry histograms of hMSCs exposed to MSN_Sr_, MSN_Sr_‐CaP, and MSN_Sr_‐CaZnP at 140 µg mL^−1^ for 6 and 24 h. c) Fluorescence intensity of labeled MSN_Sr_‐CaZnP in supernatant after 3, 7, 14, and 21 days of culture measured by a microplate reader. Positive control (Pos. ctrl) is cell culture media with 140 µg mL^−1^ labeled MSN_Sr‐_CaZnP. d) Flow cytometry histograms of MSN_Sr_‐CaZnP uptake, by hMSCs exposed to single dose and multiple doses after 3, 7, 14, and 21 days of cell culture.

Next, hMSCs internalization of fluorescently core labeled MSN_Sr_, MSN_Sr_‐CaP, and MSN_Sr_‐CaZnP was visualized using fluorescent and confocal microscopy (**Figure** **4**a and Figure S5, Supporting Information). Nanoparticles could be observed within hMSCs, where hMSC morphology remained intact (Figure 4a). Moreover, orthogonal sectioning using confocal microscopy revealed that the nanoparticles spread throughout the hMSCs and were internalized (Figure S5, Supporting Information).

hMSC uptake of MSN_Sr_, MSN_Sr_‐CaP, and MSN_Sr_‐CaZnP after 6 and 24 h was further quantitatively analyzed using flow cytometry. All three types of MSNs were internalized relatively fast after 6 h of exposure; over 95% of hMSCs were positive for nanoparticle uptake (Figure 4b, left panel). Furthermore, MSN_Sr_‐CaP and MSN_Sr_‐CaZnP showed higher uptake compared to MSN_Sr_, indicating that the CaP coating positively affected nanoparticle cell uptake. After 24 h, 83.6% (MSN_Sr_), 89% (MSN_Sr_‐CaP), and 79.5% (MSN_Sr_‐CaZnP) of hMSCs had internalized nanoparticles (Figure 4b, right panel).

Next, we set out to investigate how long‐term nanoparticle exposure, matching our hMSC osteogenic differentiation protocols of 21 days, affected hMSCs nanoparticle uptake. Since similar uptake for all three nanoparticle types was observed, MSN_Sr_‐CaZnP was chosen for this experiment. The effect of single and multiple doses (media change every 3 days) of 140 µg mL^−1^ MSN_Sr_‐CaZnP (termed MSN_Sr_‐CaZnP(s) and MSN_Sr_‐CaZnP(m), respectively) on their uptake efficiency in hMSCs was investigated using flow cytometry. The amount of MSN_Sr_‐CaZnP present in the cell culture media was also measured, and remained steady over‐time even after multiple dose exposures (Figure 4c). This indicates that the cells did not excrete the nanoparticles.

After 3 days of exposure, no significant difference in nanoparticle uptake after single or multiple dose administration could be observed (86.6% for MSN_Sr_‐CaZnP(s), and 91.5% for MSN_Sr_‐CaZnP(m)). However, after 7 days, single dose exposure resulted in a lower signal and after 14 days no significant signal could be detected using flow cytometry (Figure 4d). In contrast, hMSCs exposed to MSN_Sr_‐CaZnP every 3 days maintained a similar nanoparticle uptake signal over time (Figure 4d).

In summary, here we showed that MSNs and CaP‐coated MSNs are efficiently and rapidly taken up by hMSCs and that exposure every 3 days can maintain the nanoparticle uptake levels as observed by flow cytometry.

### 2.4 Alkaline Phosphatase (ALP) Activity

**Figure 5:**
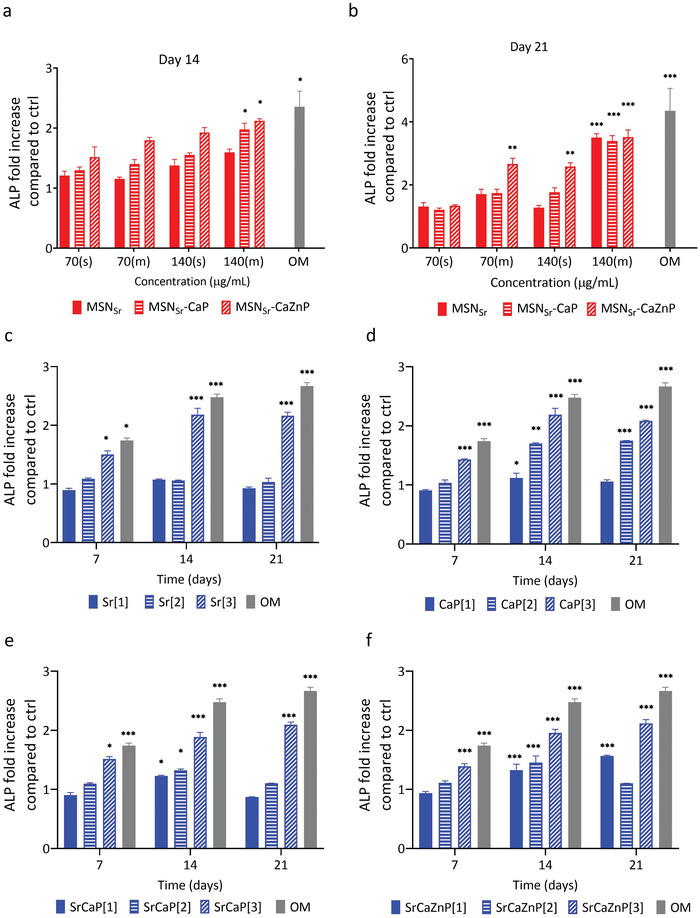
Relative ALP activity in hMSCs after exposure to a,b) MSNs or c–f) ions directly dissolved in media. hMSCs were exposed to a single dose (s) or multiple doses (m) of 70 or 140 µg mL^−1^ of MSN_Sr_, MSN_Sr_‐CaP, and MSN_Sr_‐CaZnP and ALP activity assayed after a) 14 and b) 21 days. hMSCs were further exposed to three concentrations sets of c) Sr, d) Ca and P, e) Sr, Ca, and P, and f) Sr, Ca, Zn, and P dissolved in cell culture media. Concentration set [1] consisted of ion concentration as present in multiple doses 140 µg mL^−1^ of MSN_Sr_, MSN_Sr_‐CaP, or MSN_Sr_‐CaZnP, while concentration sets [2] and [3] corresponded to ion concentrations found in literature that are known to induce osteogenic markers expression in hMSCs (Table 3). The data were normalized to hMSC DNA content. For all figures, error bars indicate one standard deviation among the triplicate. Significant difference compared to the controls are shown by * representing *p*‐values as follows; *p < 0.033; **p < 0.02; ***p < 0.001.

Next, we assessed the ability of MSN_Sr_, MSN_Sr_‐CaP, and MSN_Sr_‐CaZnP to induce enzymatic ALP activity. ALP plays a critical role in hard tissue formation by facilitating mineralization and reducing pyrophosphate concentrations, a mineralization inhibitor.^[^ ^16^ ^]^ hMSCs were exposed to single and multiple doses (70 and 140 µg mL^−1^) of the three nanoparticles in basic media (no osteogenic stimulants) and ALP activity was measured after 14 and 21 days (**Figure** **5**). In addition, hMSCs cultured in osteogenic media (OS) were included as positive control. The data were normalized for DNA content and expressed relative to ALP activity of hMSCs in basic medium.

After 14 days, a significant change in ALP activity was observed in hMSCs exposed to multiple doses of 140 µg mL^−1^ of MSN_Sr_‐CaP and MSN_Sr_‐CaZnP. In contrast, multiple dose administrations of MSN_Sr_ only led to a 1.59‐fold upregulation of ALP activity (Figure 5a).

After 21 days, hMSCs exposure to multiple doses of 140 µg mL^−1^ of all three MSNs led to a significant increase in ALP activity, (3.5‐, 3.4‐, and 3.5‐fold for MSN_Sr_, MSN_Sr_‐CaP, and MSN_Sr_‐CaZnP, respectively). Although no significant increase in ALP activity could be observed for single administrations for the three tested MSNs after 14 days, a single dose of MSN_Sr_‐CaZnP was sufficient to significantly increase ALP levels (2.58‐fold) after 21 days of exposure. In addition, multiple doses at a lower concentration (70 µg mL^−1^) of MSN_Sr_‐CaZnP also resulted in significant increased ALP activity after 21 days (2.66‐fold; Figure 5b). Overall, all three MSNs were able to induce ALP enzyme expression in hMSCs, where MSN_Sr_‐CaZnP were most effective also when applied at single and multiple lower doses.

Next, we assessed the ability of multiple doses of Ca, P, Zn, and Sr ions dissolved directly in the cell culture media to induce ALP activity in hMSCs, in order to compare its effectiveness to the nanoparticles. Similar to MSN multiple dose exposure experiments, the media was refreshed every 3 days. Three different concentrations were chosen based on our experimental settings and values found in literature. Specifically, ion concentration set [1] consisted of bioinorganics present in the same concentrations as found in 140 µg mL^−1^ of MSNs. Concentration set [2] and [3] corresponded to ion concentrations found in literature that are known to increase ALP activity in hMSCs (**Table** **3**).^[^ ^2^, ^5^ ^]^

**Table 3:** Sr, Ca, P, and Zn ions concentrations dissolved in cell culture medium

Sample code	Ion	Concentration [1]	Concentration [2]	Concentration [3]
Sr	Sr	0.723 × 10^−6^ m	25 × 10^−6^ m	200 × 10^−6^
CaP	Ca P	96.25 × 10^−6^ m 74.637 × 10^−6^ m	1.8 × 10^−3^ m 0.09 × 10^−3^ m	20 × 10^−3^ m 5 × 10^−3^ m
SrCaP	Sr Ca P	0.723 × 10^−6^ m 96.25 × 10^−6^ m 74.637 × 10^−6^ m	25 × 10^−6^ m 1.8 × 10^−3^ m 0.09 × 10^−3^ m	200 × 10^−6^ m 20 × 10^−3^ m 5 × 10^−3^ m
SrCaZnP	Sr Ca P Zn	0.723 × 10^−6^ m 96.25 × 10^−6^ m 74.637 × 10^−6^ m 9.31478 × 10^−6^ m	25 × 10^−6^ m 1.8 × 10^−3^ m 0.09 × 10^−3^ m	200 × 10^−6^ m 20 × 10^−3^ m 5 × 10^−3^ m 10 × 10^−3^ m

Addition of Sr or Ca and P at the highest tested concentration [3] resulted in increased ALP activity after 7, 14, and 21 days (Figure 5). Interestingly, when Sr, Ca, P or Sr, Ca, P, and Zn were presented together in cell medium, significant ALP induction was also observed at lower concentrations [1] and [2], albeit significantly lower (below twofold) than what was observed after exposure to the nanoparticles.

No significant ALP induction was observed when hMSCs were exposed to the concentrations as found in the MSNs (concentration [1]) (Figure 5). This indicates that the concentration of ions required to increase ALP activity in hMSCs is much lower when administered via MSN constructs compared to dissolving ions directly in the medium (Table 3). In fact, the effective concentrations required for direct ion supplementation were significantly higher compared to administration using MSNs (specifically; 276 times for Sr, 207 times for Ca, 66 times for P, and 1073 times for Zn). Thus, multiple ions administered via MSNs appear to be more effective in inducing ALP expression than multiple administration of the same ions dissolved within cell culture medium.

### 2.5 Osteogenic Gene Expression and Mineralization

Next, the effect of the MSNs on known early, middle, and late osteogenic markers were tested using quantitative real time polymerase chain reaction (qPCR) and compared to ions directly dissolved in the cell culture medium. Specifically, early osteogenic marker expression (BMP2 and RUNX2) were quantified after 7 and 14 days. RUNX2 is effective in the first stage of bone formation where mesenchymal precursors commit to the osteoblast differentiation lineage, and BMP2 is related to regulating the bone marrow matrix microenvironment and promoting osteogenic differentiation of hMSCs.^[^ ^17^ ^]^ Intermediate osteogenic marker (OPN), which participates in bone remodeling via promoting osteoclastogenesis and osteoclast activity,^[^ ^18^ ^]^ was quantified after 14 days. Finally, late osteogenic and mineralization marker (OCN),^[^ ^19^ ^]^ and matrix production (Col1)^[^ ^20^ ^]^ marker expression were quantified after 21 days. Similar to the ALP assay, hMSCs were exposed to 140 µg mL^−1^ MSNs, where a media change took place every 3 days (labeled as MSN_Sr_(m), MSN_Sr_‐CaP(m), and MSN_Sr_‐CaZnP(m)). A single dose of 140 µg mL^−1^ MSN_Sr_‐CaZnP was also included (labeled as MSN_Sr_‐CaZnP(s)), and cells in OS medium were included as positive control. Based on our previous results, we selected Sr, SrCaP, and SrCaZnP ions dissolved in the medium at the highest concentration [3] for these experiments. Importantly, basic media (without addition of osteogenic molecules) was used for all of our sample conditions.

**Figure 6:**
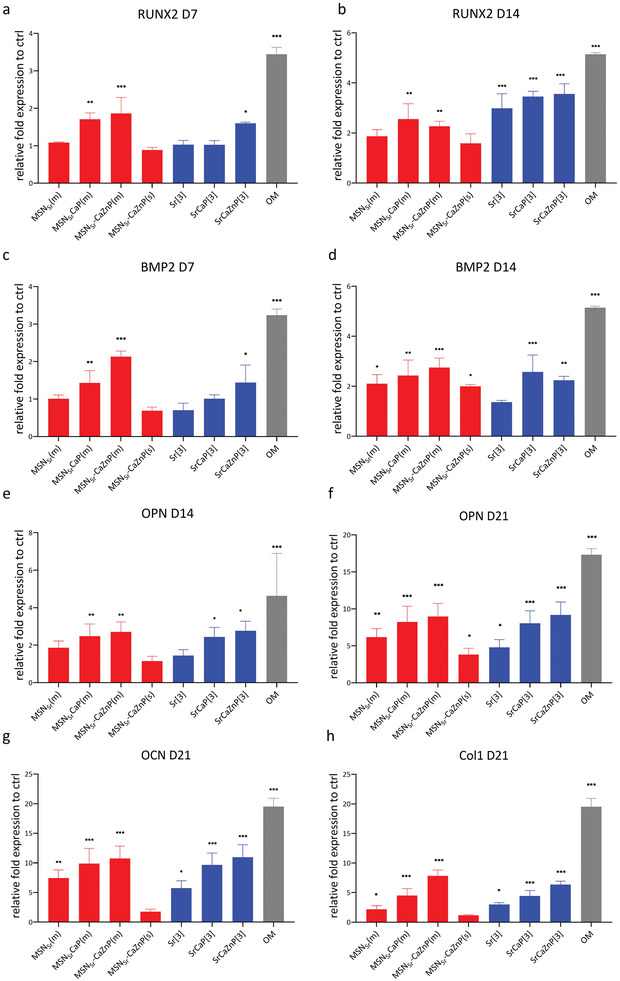
hMSC mRNA expression of RUNX2 at a) 7 and b) 14 days; BMP2 at c) 7 and d) 14 days; OPN at e) 14 and f) 21 days, OCN at g) 21 days, and Col I at h) 21 days after exposure to MSNs (MSN_Sr_(m), MSN_Sr_‐CaP(m), MSN_Sr_‐CaZnP(m), and MSN_Sr_‐CaZnP(s)) and medium dissolved ions (Sr[3], SrCaP[3], and SrCaZnP[3]) in basic conditions. The results were normalized to GAPDH as housekeeping gene and calibrated to mRNA level of the respective markers in hMSCs cultured with basic media as the control. hMSCs cultured with osteogenic medium (OM) were used as positive control. Significant difference between sample and basic control groups are shown by * representing *p*‐values as follows; *p < 0.033; **p < 0.02; ***p < 0.001.

PCR analysis showed that all tested MSNs, and their respective ions dissolved in medium at concentration [3] (Table 3) could significantly induce the expression of several osteogenic markers in hMSCs relative to the controls (**Figure** **6**a–h). Interestingly, an increased effect on osteogenic gene expression could be observed when ions were used in combination, independent of how the ions were administered.

Specifically, after 7 and 14 days, hMSCs exposed to MSN_Sr_‐CaP(m) and MSN_Sr_‐CaZnP(m) showed significantly increased RUNX2 expression (Figure 6a,b). Ion concentrations Sr[3], SrCaP[3], and SrCaZnP[3] resulted in significantly increased RUNX2 expression only after 14 days of exposure.

After 7 days of exposure, BMP2 expression was significantly upregulated for hMSCs exposed to MSN_Sr_‐CaP and MSN_Sr_‐CaZnP. After 14 days, all MSNs significantly induced BMP2 expression. Similarly, the addition for medium dissolved ions resulted in increased BMP2 expression after 14 days, except for Sr[3] (Figure 6c,d).

After 14 days of exposure, MSN_Sr_‐CaP(m) and MSN_Sr_‐CaZnP(m) also significantly induced OPN expression. While hMSCs exposed to MSN_Sr_(m) also showed an increase in OPN expression, the upregulation was relatively low compared to the other groups (1.86‐fold). After 21 days, all MSN conditions significantly increased OPN expression, where MSN_Sr_‐CaP(m) and MSN_Sr_‐CaZnP(m) resulted in the highest induction. Similarly, medium containing combinations of ions (i.e., SrCaP[3] and SrCaZnP[3]) resulted in increased OPN expression already after 14 days (Figure 6e), and all ion conditions led to upregulated OPN after 21 days of exposure (Figure 6f).

After 21 days of exposure, OCN was significantly expressed after exposure to MSNs with the exception of single administration of MSN_Sr_‐CaZnP(s). Also with this marker, increased levels were observed when a CaP layer on the MSNs was present (Figure 6g). Similarly, all conditions using ions dissolved in media led to increased OCN expression after 21 days exposure.

Finally, all MSN conditions, except for single administration of MSN_Sr_‐CaZnP, led to increased expression of Col1 (Figure 6h). Significantly increased Col1 gene expression was also observed for conditions when multiple ions were used.

**Figure 7:**
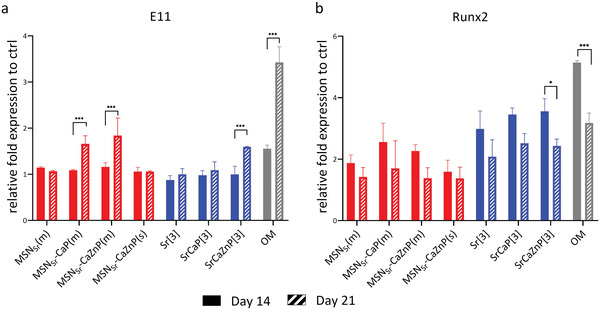
hMSC mRNA expression of a) Runx2 and b) E11 at 14 and 21 days after exposure to nanoparticles composites (red) and ions (blue). All conditions were cultured in basic cell culture media. The results were normalized to GAPDH as housekeeping gene and calibrated to mRNA level of the respective markers in hMSCs cultured with basic media as the control. hMSCs cultured with osteogenic medium (OM) were used as positive control (gray). Significant differences between day 14 and day 21 samples are shown by * representing *p*‐values as follows; *p < 0.033; **p < 0.02; ***p < 0.001.

To assess whether our nanoparticles could also induce the transition from osteoblast to osteocyte, gene expression of osteocyte marker podoplanin (E11) was evaluated. Podoplanin is a known marker that is upregulated during the embedding of osteocytes in a mineralized matrix.^[^ ^21^ ^]^ We compared podoplanin induction to RUNX2 expression after 14 and 21 days (**Figure** **7**). Significantly increased podoplanin expression could be observed after 21 days of exposure to MSN_Sr_‐CaP(m), MSN_Sr_‐CaZnP(m), and SrCaZnP[3]. Conversely, a trend toward a lower expression of Runx2 was observed after 21 days of culture, although not significant. These data indicate that the overall population is transitioning to osteocytes in conditions when multiple ions are present and after longer exposure times.

**Figure 8:**
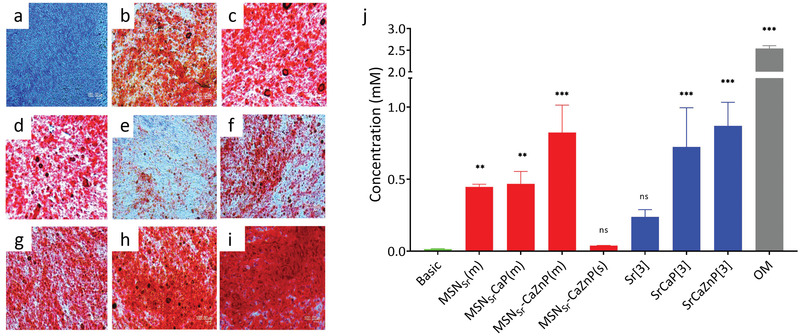
Light microscopy images of mineral formation by hMSCs (*n* = 3) when cultured in a) basic medium, b) medium containing MSN_Sr_(m), c) MSN_Sr_‐CaP(m), d) MSN_Sr_‐CaZnP(m), e) MSN_Sr_‐CaZnP(s), f) Sr[3], g) SrCaP[3], and h) SrCaZnP[3] compared to i) osteogenic medium (OM). Calcium minerals were stained with Alizarin Red S. j) Quantification of calcium content at day 28 was performed using a de‐staining method. Significant difference between samples and control groups are shown by * representing *p*‐values as follows; *p < 0.033; **p < 0.02; ***p < 0.001, ns means nonsignificant.

Next, we investigated the effect of the nanoparticles and dissolved ions on hMSC mineralization using Alizarin red staining. hMSCs cultured in basic and mineralization media were used as negative and positive controls, respectively. hMSCs cultured in basic media did not show any signs of mineralization (**Figure** **8**a). In contrast, hMSCs exposed to multiple doses of the three types of MSNs showed high mineralization (Figure 8b–i). All three ion conditions also resulted in significant mineralization. Exposure to a single dose of MSN_Sr_‐CaZnP was not sufficient to promote significant mineralization in hMSCs (Figure 8e,j).

Furthermore, the amount of calcium deposited by hMSCs exposed to MSN_Sr_(m) and MSN_Sr_‐CaP(m) was similar (0.445 × 10^−3^ and 0.467 × 10^−3^ m, respectively), while hMSCs cultured with MSN_Sr_‐CaZnP(m) showed highest amount of calcium deposition (0.823 × 10^−3^ m; Figure 8j). In contrast, single administration of MSN_Sr‐_CaZnP resulted in only 0.0388 × 10^−3^ m of calcium deposition. Thus, multiple exposures to MSN_Sr_, MSN_Sr_‐CaP, and MSN_Sr‐_CaZnP promoted the mineralization process during osteogenesis of hMSCs, where MSNs with a CaP and CaZnP layer showed the highest effect. This was also observed for the hMSCs exposed to ions dissolved in medium, where high mineral deposition was observed for SrCaP[3] and SrCaZnP[3] (0.722 × 10^−3^ and 0.869 × 10^−3^ m, respectively) and relatively low mineral deposition for hMSCs exposed to Sr[3] (0.237 × 10^−3^ m).

## 3 Discussion

In this work, we developed MSNs incorporating Sr and coated with a pH‐sensitive CaP or CaZnP layer. Doping ceramic nanoparticles and scaffolds with bioactive ions is a popular strategy to improve their bone regenerative capabilities. Many examples are known especially in the bioactive glass field where doping with ions such as Sr, Zn, and copper has resulted in materials with improved bone regenerative capabilities.^[^ ^2a^ ^]^ While there are several studies using MSNs to deliver known osteogenic stimulants such as dexamethasone and BMP‐2,^[^ ^22^ ^]^ the use of MSNs for ion delivery is limited to several reports.^[^ ^23^ ^]^ In these studies, the ions are doped within the silica matrix, where particle degradation is needed to allow ion release. There are no studies looking into using MSNs as stable carriers for multiple ion delivery, as we have presented here. To prepare the nanoparticles, we surface grafted CaP onto MSNs to act both as pH‐sensitive gating system to prevent Sr release and to facilitate delivery of CaP ions upon nanoparticle uptake in hMSCs. Previously, it has been reported that MSNs are actively taken up by MSCs via endocytosis,^[^ ^13^ ^]^ which involves the formation of acidic endosomal vesicles containing an average pH of 5. CaP solubility is dependent on its crystallinity and pH and CaP solubility dramatically increases below pH 6.0.^[^ ^24^ ^]^ Several other examples exist where CaP surface‐modified MSNs have been used for pH‐responsive drug delivery of anticancer drugs.^[^ ^25^ ^]^ Our method used direct CaP precipitation on carboxylic acid surface‐modified MSNs, similar to a previously published method.^[^ ^25b^ ^]^ We modified the procedure to also create carboxyl group within the mesopores to increase Sr ion loading capacity of the MSNs. In addition, we modified the protocol to also introduce Zn on the CaP surface coating, as Zn is a popular additive to promote bone regeneration in dentistry and orthopedic applications.^[^ ^26^ ^]^

The developed MSNs were efficiently taken up by hMSCs. Our results further showed that CaP coating of MSNs increases their uptake. This is in line with other studies showing that various nanoparticle structural features including size, shape, and surface chemistry and charge effect nanoparticle cell internalization.^[^ ^27^ ^]^ Several studies suggest that the majority of MSN uptake by hMSCs goes via clathrin‐mediated endocytosis but also involves unknown pathways.^[^ ^13^ ^]^ The MSC uptake mechanism of CaP nanoparticles seems to involve not only clathrin‐mediated endocytosis but also ATP‐dependent endocytosis.^[^ ^28^ ^]^

After single dose exposure, the nanoparticle signal disappeared within 7 days, however, there was no increase in fluorescence in the cell culture media indicating that the cells did not excrete the nanoparticles. Although there are only a few studies that have looked into the long‐term uptake of MSNs in MSCs, our data are in line with what we and others have previously reported. Huang et al. demonstrated that FITC‐labeled MSNs could still be observed by confocal microscope 21 days after single exposure to hMSCs. However the percentage of positively labeled hMSCs could only be detected up to 7 days via flow cytometry indicating that the particle number per cell reduced over‐time due to cell proliferation.^[^ ^29^ ^]^ In our previous study, we observed that the MSN signal was halved after every cell passage, resulting in the loss of signal in flow cytometry after about 15 days.^[^ ^14a^ ^]^ Here, we showed that when we expose the hMSCs to multiple doses in 3 day intervals, the level of nanoparticle uptake can be maintained over 21 days when observed using flow cytometry. Moreover, although we did not test this condition, our data indicate that administration every 7 days would also be sufficient to maintain nanoparticle levels.

Taken together, our ALP activity, gene expression, and mineralization data demonstrated that MSNs incorporating Sr and coated with CaZnP layer (MSN_Sr_‐CaZnP) were most osteogenic. MSN_Sr_‐CaZnP could significantly induce early, middle, and late osteogenic markers in hMSCs at all tested time points and led to pronounced mineralization. MSNs containing only Sr ions (MSN_Sr_) were least effective, indicating that there is an additive effect when administering multiple ions within a single nanoparticle construct. This additive effect was also observed in the conditions where ions were added directly to the cell culture media; also here Sr[3] was the least effective condition, whereas SrCaZnP[3] showed the strongest osteogenic gene induction and most pronounced mineralization. The effects of combining bio‐inorganic ions have only limitedly been explored. Several studies have shown that incorporating Sr, Si, or Zn ions into CaP scaffolds improves bone formation capabilities compared to scaffolds without additional ions.^[^ ^8^ ^]^ Mao et al. compared the effect of Sr and Si media extract from *β*‐TCP to Sr and Si ions alone on osteoblast cells. It was found that Sr and Si ions possessed synergistic effects by upregulating the expression of osteogenic gene OCN and angiogenic gene VEGF in rBMSCs‐OVX cells.^[^ ^8a^ ^]^ Jia et al. have previously shown that ALP, OCN, and Col I were upregulated when mouse osteoblasts were exposed to a combination of Zn and Sr released from Zn‐Sr alloy submerged in media compared to pure Zn.^[^ ^8b^ ^]^ To our knowledge, our study is the first to use a single nanoparticle construct to deliver a combination of SrCaP and SrCaZnP ions to hMSC, and to demonstrate that this has positive effects on osteogenic marker expression.

Although the ions dissolved in the cell culture medium had a similar effect on the osteogenic gene expression in hMSCs as the ion‐incorporated MSNs, the concentrations required to obtain this effect by directly dissolving ions in the medium were significantly higher. Indeed, the cumulative ion concentration present in the nanoparticles was 134 (P), 208 (Ca), 277 (Sr), and 538 (Zn) times lower compared to respective ions when dissolved in the cell culture medium (Table 3). When exposing hMSCs to the same effective ion dose in the cell culture medium as was present in the ions‐functionalized MSNs, no significant ALP activity was observed (Figure 5). This difference may be due to different uptake routes: where nanoparticle internalization in hMSCs is known to involve endocytosis, ions are generally taken up via ion channels. By surface‐coating the particles with a CaP coating that is degradable only at lower pH, the ion dissolution only takes place at low pH values such as those found in endosomes, while they remain stable at pH 7. This pH‐responsive mechanism likely decreased their effective dose by preventing unwanted release of ions when not internalized. Importantly, our MSNs could induce osteogenesis in hMSCs without the presence of any other osteogenic stimulants in the media (e.g., under basic conditions). Another advantage of using our MSN composites is that the administration dose and frequency can potentially be reduced. Even though we did not test other conditions, our flow cytometry data suggest that administering the nanoparticles every 7 days should also be effective. This advantage combined with lower effective doses, make our developed MSN‐CaP promising constructs for use in bone regeneration applications.

## 4 Conclusions

Bioinorganic ions are promising additives in bone regenerative biomaterial‐based strategies to stimulate bone and blood vessel formation. However, local ion release and dosage control are critical to prevent adverse side effects. In this study, we demonstrated that silica‐based nanoparticles (MSNs) incorporating Sr ions and coated with CaP or CaZnP layers are promising ion carriers for bone regenerative strategies. The CaP or CaZnP coat acted as a pH‐sensitive gating system with rapid ion release when in acidic conditions.

Our developed MSNs could efficiently induce the expression of early, middle, and late osteogenic markers in the absence of other stimulators of osteogenic differentiation. While all the MSNs tested here had a stimulatory effect, MSNs containing CaZnP surface coating were most osteogenic while MSNs without CaP coat (MSN_Sr_) were least effective, indicating an additive effect when delivering multiple bioinorganic ions using MSNs. To our knowledge, our study is the first to use a single nanoparticle construct to deliver a combination of Sr, Ca, Zn, P ions, and to demonstrate that this has positive effects on osteogenic marker expression in hMSCs. Furthermore, our MSNs promoted osteogenic marker expression in hMSCs at a much lower dose compared to adding the same ions but directly dissolved in cell culture medium, likely due to different cell internalization uptake routes. As such, the developed MSNs represents a promising strategy to decrease ion dosage and effectively induce hMSC osteogenesis. A further in‐depth understanding of how ion dosing and possible synergy influences not only osteogenesis but also other regenerative processes such as angiogenesis could potentially maximize their use in bone regeneration approaches.

The type of MSNs developed here could be easily applied in injectable biomaterials and printable scaffolds used for bone regeneration purposes without impairing their mechanical properties. Additional advantages of using MSNs is that they allow ion release and dosage control, and they can be surface modified to target cells. Considering these advantages combined with lower effective and less required administration doses, the MSNs developed here represent promising constructs for use in bone regeneration applications.

## 5 Experimental Section

### Materials

Tetraethyl orthosilicate (TEOS), 3‐merCaPi topropyl triethylsilane (MPTES), triethanolamine (TEA), APTES, cetyltrimethylammonium chloride (CTAC), ammonium fluoride, hydrochloric acid (37 V/V%), ammonium nitrate, *N*,*N*‐dimethylformamide (DMF), phosphate‐buffered saline (PBS), fetal bovine serum (FBS), ascorbic acid, bis[*N*,*N*‐bis(carboxymethyl)aminomethyl] were purchased from Sigma Aldrich GmbH (Germany). Absolute ethanol, paraformaldehyde (PFA), Triton X‐100, bovine serum albumin (BSA), Tween‐20, and Alizarin Red S (sodium alizarin sulfonate), minimum essential medium alpha (*α*MEM), L‐glutamine, and trypsin were purchased from Fisher Scientific (The Netherlands). Penicillin and streptomycin were purchased from Gibco Life Technologies (US). Concentrated nitric acid 60 V/V% (HNO_3_), hydrochloric acid 37 V/V% (HCL), element standards of Ca, Si, P, Sr, Zn, and scandium (Sc) for ICP‐MS were also purchased from VWR (US).

### Synthesis of Mesoporous Silica Nanoparticles (MSNs)

Synthesis of MSNs with thiol functional groups in the particles’ core and functional amine groups at the particles’ surface was based on the co‐condensation method as previously reported.^[^ ^13a^ ^]^ Further details on MSN synthesis and characterization can be found in the Supporting Information (Section S1). The amine groups in the MSN pores and surface were further modified to obtain carboxylic acid groups, which allowed positive ion deposition. Carboxyl‐functionalized MSN nanoparticles (MSN‐COOH) were obtained as follows; 3 g of succinic anhydride was added in 20 mL DMF, and allowed to fully dissolve for 30 min at room temperature. Subsequently, 0.2 g of MSNs were dispersed in 30 mL DMF, and the suspension was added into the above solution under magnetic stirring. The reaction was kept at 60 °C. After stirring for 48 h, the suspension was collected by centrifugation and extensively washed with absolute ethanol. MSNs were stored at −20 °C until further use.

### Strontium Ion Loading and Calcium, Phosphate, Zinc Composite Layer Formation

To create MSNs containing Sr ions (MSN_Sr_), MSN‐COOHs were suspended in 10 × 10^−6^ m SrNO_3_ in milliQ water overnight. The suspension was collected by centrifugation and washed once with milliQ water. To synthesize calcium phosphate‐coated MSNs (MSN_Sr_‐CaP) and calcium phosphate, zinc‐coated MSNs (MSN_Sr_‐CaZnP), 100 mg of MSN_Sr_ was re‐suspended in 100 mL milliQ water adjusted to pH 10 by NH_4_NO_3_. 200 µL of 4 × 10^−6^ m (NH_4_)_3_PO_4_ was added to the mixture and allowed to react for 30 min. After 30 min, 200 µL of 6 × 10^−6^ m calcium nitrate for MSN_Sr_‐CaP or 6 × 10^−6^ m calcium nitrate and zinc (10:1 molar ratio) solution for MSN_Sr_‐CaZnP was added and allowed to react for 30 min. Calcium and phosphate ion addition was repeated two more times to achieve nanoparticle sizes of roughly 100 nm. The nanoparticles were then collected by centrifugation and washed with absolute ethanol. MSN_Sr_‐CaP and MSN_Sr_‐CaZnP were stored at −20 °C until further use.

### Characterization of the MSNs

The MSNs were characterized for their morphology and size using transmission electron microscopy (TEM FEI Tennai G2 Spirit BioTWIN iCorr (G0.201)). For this, 5 µL of 0.3 µg mL^−1^ MSNs suspended in ethanol were dropped onto the gold TEM grids and dried overnight before imaging.

The total ion composition in the composite nanoparticles was determined by ICP‐MS (iCaP Q, Thermo Scientific). For this, MSN_Sr_, MSN_Sr_‐CaP, and MSN_Sr_‐CaZnP were digested with aqua regia (nitric acid:hydrochloric acid in a molar ratio of 1:3) until completely dissolved. Digested MSNs were diluted 1:10 in aqueous 1% HNO_3_ containing 20 ppb Sc as internal standard and analyzed using He as collision gas in normal mode. Element quantification was based on calibration with element standards of Ca, Si, P, Sr, and Zn. Results were expressed as percentage of the total element presented for each type of nanoparticle. XRD of all synthesized MSNs was collected using a Bruker D2 Phaser diffractometer (Bruker) using Cu K*α* radiation (*λ* = 1.5406 Å) in the range of 6° ≤ 2*Θ* ≤ 50° in increments of 0.02° and an integration time of 0.5 s.

### Degradation Study

The degradation of MSN_Sr_, MSN_Sr_‐CaP, MSN_Sr_‐CaZnP was studied for up to 6 days at pH 5 and pH 7.4 at a concentration of 1 mg mL^−1^ in cacodylate buffer. After 1, 3, 6, 24, 72, and 144 h, leftover solid and supernatants were separated by centrifugation and stored at −20 °C for subsequent elemental analysis. The content of Ca, P, and Si was assessed by ICP‐MS (Thermo Scientific). None degraded solid were digested with aqua regia until completely dissolved. Both digested and supernatant were diluted 1:10 in aqueous 1% HNO_3_ containing 20 ppb Sc as internal standard and analyzed using He as collision gas in normal mode. Element quantification was based on calibration with element standards of Ca, Si, P, Sr, and Zn. Results were expressed as percentage of the total element presented in each type of nanoparticles.

### In Vitro Cell Culture

hMSCs (PromoCell) of passage 3 were expanded in *α*MEM with addition of 10% FBS, 2 × 10^−3^ m L‐glutamine, 0.2 × 10^−3^ m ascorbic acid, 100 U mL^−1^ penicillin, and 100 mg mL^−1^ streptomycin at 37 °C, 5% CO_2_ in a humidified atmosphere. Cells of passage 4 were used for the experiments. 1 mL of cells suspended in medium (5000 cells per cm^2^) were seeded in each well of 12‐well cell culture plates. Cells were left to adhere for 24 h before change of media.

### Cell Uptake of Nanoparticles

Flow cytometry was performed to quantitatively assess the uptake of the MSNs by hMSCs. For short‐term flow cytometry uptake study, hMSCs were seeded in 12‐well plates at 5000 cells per cm^2^ density. ATTO‐633‐maleimide‐labeled MSNs (labeling procedure can be found in the Supporting Information) were suspended in cell culture media at a concentration of 70 µg mL^−1^ and exposed to the hMSCs when they had reached 80–90% confluence. Flow cytometry was performed after 6 and 24 h of MSN exposure. To prepare the samples, cells were washed with PBS, trypsinized, and re‐dispersed in culture medium. Cell suspensions were then centrifuged and re‐dispersed in 300 µL PBS and kept on ice for flow cytometry analysis (BD Accuri C6). A total of 10 000 cells (gated) were collected for each measurement. FlowJo version 10 was used for data analysis.

Cellular uptake of MSNs was also analyzed by immunohistochemical staining. hMSCs were seeded at a density of 3000 cells cm^−2^ on a plasma‐treated cover slide. At 80–90% confluency, hMSCs were exposed to ATTO‐647‐maleimide‐labeled MSNs for 6 and 24 h. After exposure, cells were fixed with 4% paraformaldehyde (PFA). Prior to staining, cells were washed once with PBS and permeabilized with Triton X‐100 (0.01% vol/vol in PBS) for 10 min at room temperature followed by washing three times with PBS. Samples were then incubated for 60 min in blocking buffer (4% BSA and 0.05% vol/vol Tween‐20 in PBS) at room temperature. To visualize actin bundles, cells were stained with Alexa FluorTM 488 phalloidin (1:200 in PBS; Thermo Fisher Scientific) for 30 min at room temperature, followed by washing three times with PBS. To visualize cell nuclei, samples were incubated for 6 min with 4′,6‐diamidin‐2‐phenylindol (DAPI, 1:70 in PBS; Sigma Aldrich) at room temperature, washed three times with PBS, and mounted with Dako (Sigma Aldrich). Cells were imaged with a Nikon Eclipse Ti‐E microscope (Nikon Instruments Europe BV, the Netherlands) using an oil objective. To further visualize internalization of MSNs without membrane permeabilization, cellular uptake of MSNs was analyzed by laser scanning confocal microscope. hMSCs were seeded at a density of 2000 cells cm^−2^ on a 6 cm^2^ glass bottom cell culture well. At 80–90% confluency, hMSCs were exposed to ATTO‐488‐maleimide‐labeled MSNs for 6 h. Cells were stained with CellMask Deep Red Plasma membrane stain (1:800 in PBS; Thermo Fisher Scientific) and DAPI (1:100 in PBS; Sigma Aldrich). Images were captured by Nikon A1R+ laser scanning confocal microscope (Nikon, Japan). Z‐stack scans were used to end sure internalization of MSNs. Images were further processed and merged using ImageJ.

For long‐term flow cytometry uptake study, hMSCs were cultured and exposed to MSNs similar to short‐term experiments. However, 140 µg mL^−1^ of labeled MSNs in media were used and media were refreshed every 3 days. MSNs were freshly added into the media just before the media change. At 1, 3, 7, 14, and 21 days of culturing, samples were collected for flow cytometry analysis.

### Cytotoxicity

The cytotoxicity of MSN_Sr_, MSN_Sr_‐CaP, MSN_Sr_‐CaZnP at concentrations ranging from 70 to 1000 µg mL^−1^, was determined using the MTT assay according to manufacturer's protocol (Sigma‐Aldrich). hMSCs were seeded in 12‐well plates at 5000 cells per cm^2^ density. As hMSCs reached 80–90% confluence, they were exposed to MSNs at concentrations ranging from 35 to 1000 µg mL^−1^. Cytotoxicity was assessed after 24 and 72 h of exposure. Amount of dissolved formazan was determined immediately using a microplate reader (BIO‐RAD microplate reader‐550) at absorbance wavelength of 570 nm. Wells with complete medium, MSNs, and MTT reagent, without cells were used as blanks.

### Alkaline Phosphatase Assay

Osteogenic differentiation was evaluated by measuring ALP levels at days 14 and 21 of culture. CyQuant Cell Proliferation Assay Kit (Thermo Fisher Scientific) was used to measure DNA content for normalization of ALP levels. To measure ALP levels and DNA content, cells were lysed with cell‐lysis buffer (provided with the kit, 1:20 in PBS) containing 0.1% vol/vol RNAse A (Thermo Fisher Scientific) and three cycles of freezing–thawing at −80 °C. First, medium was discarded, and cell layers were washed once with PBS. After freezing–thawing for 30 min, RNAse lysis buffer was added to each well, and samples were frozen–thawed for two cycles of 30 min each. Once thawed, more RNAse lysis buffer was added, and samples were sonicated for 5 min. To assure complete lysis of the cells, samples were then incubated for 60 min at room temperature.

ALP activity was quantified using CDP‐star solution (ready‐to‐use, Sigma Aldrich). Cell lysate was incubated 1:5 with the reagent for 30 min in the dark at room temperature in a white‐bottom, 96‐well plate. Using a microplate reader (BIO‐RAD microplate reader‐550), ALP values were normalized with total DNA content per sample and expressed as an x‐fold increase compared to hMSCs cultured on plastic with nonosteogenic medium. For the quantification of total DNA content, cell lysate was mixed with GR‐dye solution (provided in the CyQuant kit, 1:200 in lysis buffer) according to the supplier's instructions. After 15 min, the fluorescent signal was measured with a spectrophotometer at 520 nm. Absolute DNA amounts were calculated using the standard curve prepared following the supplier's instructions.

### RNA Extraction and Gene Expression (qPCR) Assay

Total RNA was isolated from hMSCs via the Trizol method. RNA was further purified using RNA isolation kit (Bioline ISOLATE II RNA Mini Kit). RNA was collected in RNAse‐free water and the total concentration was measured using nano‐drop (Thermo Scientific NanoDrop). The cDNA of the cultures were then prepared using an iScript kit (Bio‐Rad) according to the manufacturer's protocol and kept in RNAse‐free water to be used for qPCR. The qPCR measurement (Bio‐Rad equipment) was performed using Syber Green I master mix (Invitrogen) using primer sequences (Sigma) as listed in **Table** **4**. Expression of osteogenic marker genes was normalized to GAPDH levels as housekeeping gene and basic fold indications were calculated by using ΔΔCT method. hMSCs not exposed to nanoparticles were used as controls. All the conditions were done with biological triplicate (*n* = 3).

**Table 4:** Primer sequence of the osteogenic genes investigated

Gene	Primer sequences
GAPDH (housekeeping gene)	5′‐CCATGGTGTCTGAGCGATGT 5′‐CGCTCTCTGCTCCTCCTGTT
RUNX 2	5′‐GGAGTGGACGAGGCAAGAGTTT 5′‐AGCTTCTGTCTGTGCCTTCTGG
Bone morphogenetic protein 2 (BMP2)	5′‐GCATCTGTTCTCGGAAAACCT 5′‐ACTACCAGAAACGAGTGGGAA
Osteopontin (OPN)	5′‐CCAAGTAAGTCCAACGAAAG 5′‐GGTGATGTCCTCGTCTGTA
Osteocalcin (OCN)	5′‐CGCCTGGGTCTCTTCACTAC 5′‐TGAGAGCCCTCACACTCCTC
Collagen I (COL I)	5′‐GATTCCCTGGACCTAAAGGTGC 5′‐AGCCTCTCCATCTTTGCCAGCA
Podoplanin (E11)	5′‐GTGTAACAGGCATTCGCATCG 5′‐TGTGGCGCTTGGACTTTGT

### Mineralization Assay

Calcium deposition of hMSCs was assessed by Alizarin Red S (sodium alizarin sulfonate) staining to visualize mineralization. After 28 days of culture, cells were fixed with 4% PFA, and calcium deposits were made visible by Alizarin Red S staining. Alizarin Red S was dissolved in bi‐distilled water at a concentration of 22 mg mL^−1^. Once the solid was completely dissolved, pH was adjusted to 4.2 with ammonium hydroxide. Prior to staining, cells were rinsed once with PBS followed by washing twice with bi‐distilled water. Alizarin Red solution was added to samples and then the samples were incubated for 15 min at room temperature followed by washing thrice with bi‐distilled water. Staining was assessed by visual inspection.

A quantification method was carried out using a de‐staining method with 10% w/v cetylpyridinium chloride (CPC) (Sigma) in 10 × 10^−3^ m sodium phosphate (pH 7.0), adding 1 mL of CPC solution per well. After incubating for 15 min at room temperature under constant shaking, 10 µL from the extracted stain was transferred to a 96‐well plate and diluted tenfold with CPC solution. The violet colored supernatant was read with a microplate reader (CLARIOstar Multimode Microplate Reader) at 555 nm.

### Statistical Analysis

For statistical comparisons for biocompatibility and ALP activity analyses, a two‐way analysis of variance (two‐way ANOVA) was used followed by Turkey's multiple comparison. For gene expression analysis, a two‐way ANOVA was performed followed by Dunnett's multiple comparison post‐hoc test. For all figures, error bars indicated one standard deviation and for the *p*‐values, the following applied: *p < 0.033; **p < 0.02; ***p < 0.001

## Conflict of Interest

The authors declare no conflict of interest.

## Supporting information

Supporting Information

## Data Availability

The data that support the findings of this study are available from the corresponding author upon reasonable request.
