# Supplementary material for: Calcium Phosphate‐Coated and Strontium‐Incorporated Mesoporous Silica Nanoparticles Can Effectively Induce Osteogenic Stem Cell Differentiation
Source: Adv Healthc Mater. 2021 Nov 25;11(4):2101588. doi: 10.1002/adhm.202101588 (PMC11468810; doi:10.1002/adhm.202101588)
Supplement: Supplementary file 1 — Supporting Information [file ADHM-11-2101588-s001.pdf]

ADVANCED HEALTHCARE MATERIALS

# Supporting Information

for *Adv. Healthcare Mater.*, DOI: 10.1002/adhm.202101588

# Calcium phosphate coated and strontium incorporated mesoporous silica nanoparticles can effectively induce osteogenic stem cell differentiation

*Pichaporn Sutthavas, Zeinab Tahmasebi Birgani, Pamela Habibovic, Sabine van Rijt*[*]

Supporting information

# Calcium phosphate coated and strontium incorporated mesoporous silica nanoparticles can effectively induce osteogenic stem cell differentiation

*Pichaporn Sutthavas*[1], *Zeinab Tahmasebi Birgani*[1], *Pamela Habibovic*[1], *Sabine van Rijt*[1*]

[1]Department of Instructive Biomaterials Engineering, MERLN Institute for Technology-Inspired Regenerative Medicine, Maastricht University, P.O. Box 616, 6200 MD Maastricht, the Netherlands

* Corresponding author:
Sabine van Rijt, PhD
Assistant Professor
Department of Instructive Biomaterials Engineering
MERLN Institute for Technology-Inspired Regenerative Medicine, Maastricht University
E-mail: s.vanrijt@maastrichtuniversity.nl

## Synthesis of core-labelled MSN

To synthesize MSNs with amines on the surface, a mixture of 1.63 g TEOS (7.82 mmol), MPTES (112 mg, 0.48 mmol) and 14.3 g TEA (95.6 mmol) was heated to 90 °C under static conditions (Solution 1). Solution 2 included 100 mg ammonium fluoride (2.70 mmol) dissolved in a solution of 2.41 ml CTAC (1.83 mmol, 25% (wt) in $H_2O$) and 21.7 ml bi-distilled water (1.21 mmol) by heating to 60 °C. Solution 2 was rapidly added to solution 1, and the mixture was stirred vigorously at 700 rpm for 20 min while left to cool. Then, 138.2 mg TEOS (0.922 mmol) was added in four equal increments (34.55 mg each) every 3 minutes. The solution was then left stirred overnight at room temperature. The particles were then collected by centrifugation at 7800 rpm for 20 min and washed once with ethanol. Template extraction was performed by dispersion into an ammonium nitrate in ethanol solution (2 g NH4NO3 in 100 ml ethanol) and refluxed for 45 minutes at 90 °C. MSNs were collected by centrifugation and washed with ethanol before further template extraction in 100 ml of a 3.7% hydrochloric acid solution in ethanol for 45 minutes at 90 °C. MSNs were collected by centrifugation, washed twice with ethanol and stored in suspension at -20 °C. The thiol groups functionalized core of the MSNs were labelled with ATTO-647-Maleimide to create fluorescent core labeled MSNs.

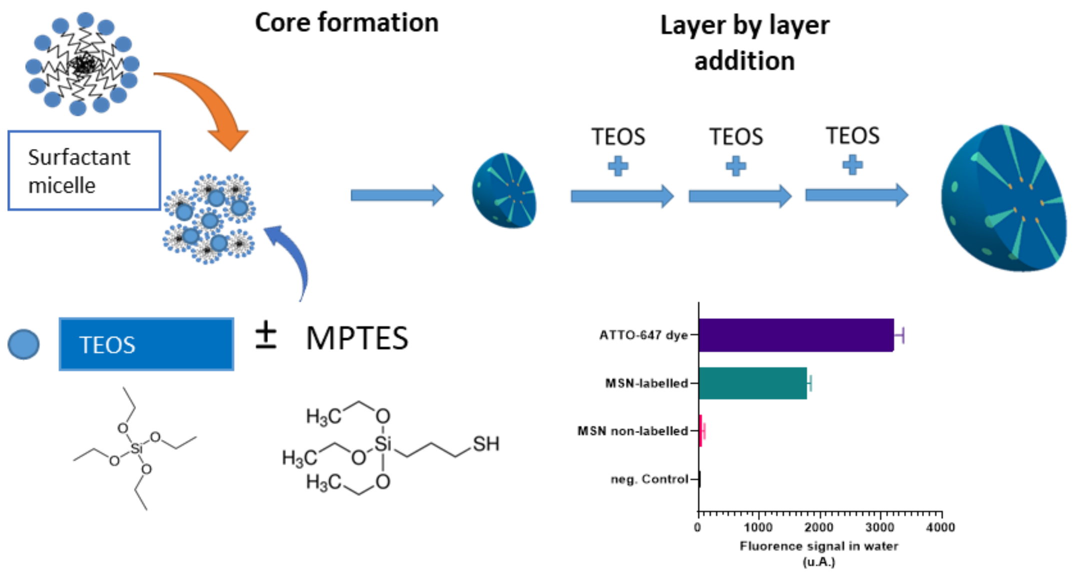


Figure S1: Synthesis scheme of core-labelled MSN. MSN labelling with ATTO-647-Maleimide confirmed core functionalization with thiols (lower right graph).

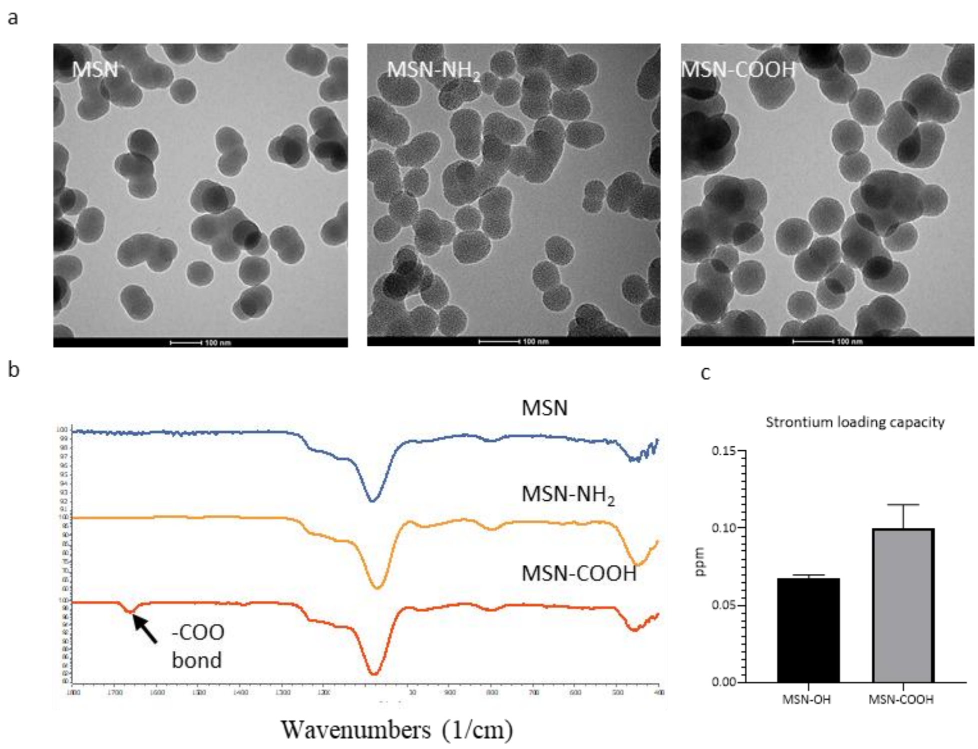


Figure S2: Characterization of MSN, MSN-$NH_2$ and MSN-COOH. a) TEM images show spherical-shaped porous structure for all nanoparticles. b) FTIR spectra of MSN, MSN-$NH_2$ and MSN-COOH. c) Strontium loading capacity of MSN-OH and MSN-COOH when MSNs of the same amount were incubated with $SrNO_3$ aqua-solution (5 mM) overnight. Quantitative analysis of Sr were preform by ICP-MS.

Table S1; Hydrodynamic size, polydispersity index (Pdi) and zeta potential of MSN, MSN-$NH_2$ and MSN-COOH in ethanol measured by DLS.

| Sample | Hydrodynamic size in ethanol (nm) | Pdi | Zeta potential |
|---|---|---|---|
| MSN-OH | 188±1.47 | 0.107 | -20.6±0.13 |
| MSN-$NH_2$ | 195.8±0.56 | 0.09 | +19.6±0.31 |
| MSN-COOH | 203±2.1 | 0.27 | -17.7±0.2 |

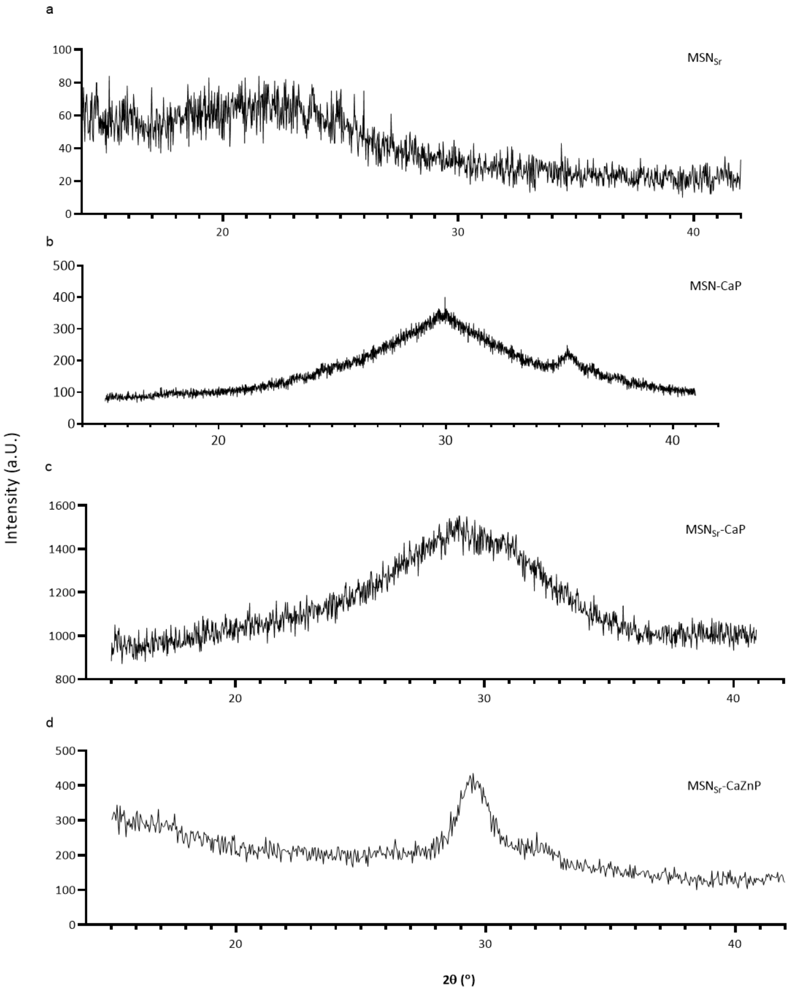


**Figure S3**. XRD patterns of $MSN_{Sr}$, $MSN_{Sr}$-CaP, MSN-CaP, and $MSN_{Sr}$-CaZnP displayed amorphous non-crystallization of all nanoparticles.

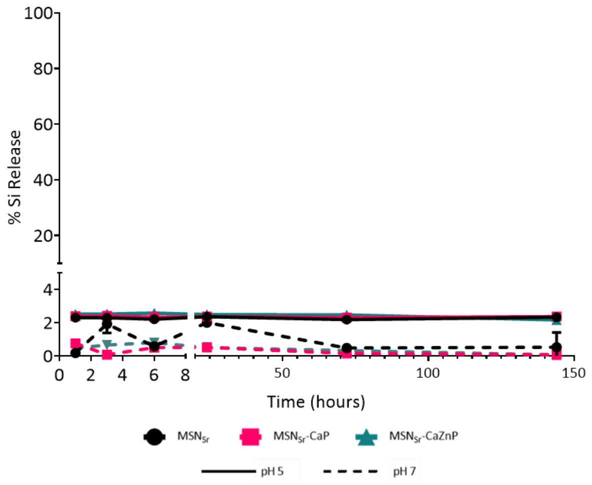


Figure S4: Silica ions release profiles of $MSN_{Sr}$ (black), $MSN_{Sr}$-CaP (red) and $MSN_{Sr}$-CaZnP (green) in cacodylate buffer with pH 5 (solid lines) and 7.4 (dashed lines) analysed by ICP-MS.

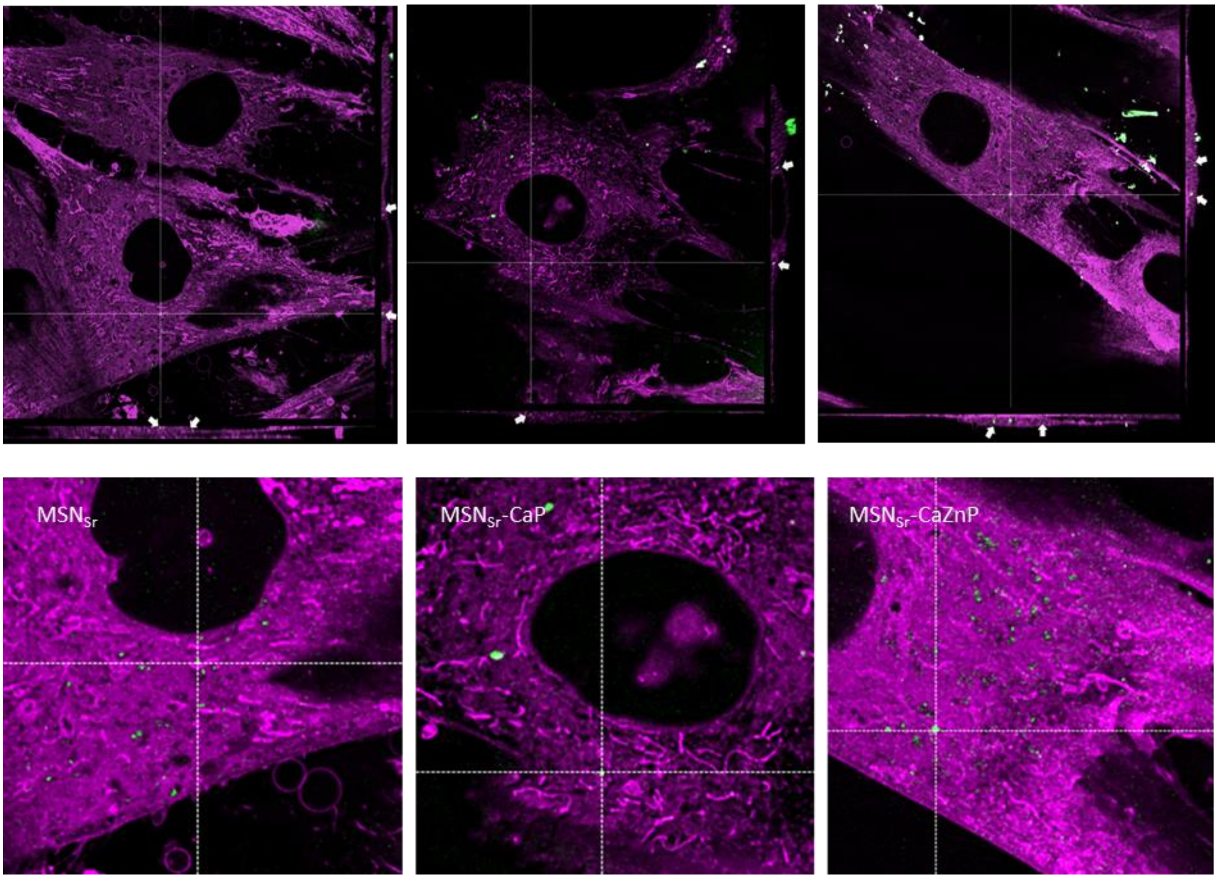


Figure S5: Confocal microscopy images of hMSCs exposed to 140 µg/ml (a) $MSN_{Sr}$, (b) $MSN_{Sr}$-CaP and (c) $MSN_{Sr}$-CaZnP for 6 hours. Cells were washed with PBS 3 times to remove non-internalized MSNs. Cell bodies were labelled with CellMask (in purple). Core labelled MSNs are shown in green. Arrows in orthogonal sections indicate the MSNs located within the cell body and not on the surface of cell membrane. Lower panel illustrates a magnification of the cells showing nanoparticle distribution in the cells.
